# Tumour targeting and radiation dose of radioimmunotherapy with ^90^Y-rituximab in CD20+ B-cell lymphoma as predicted by ^89^Zr-rituximab immuno-PET: impact of preloading with unlabelled rituximab

**DOI:** 10.1007/s00259-015-3025-6

**Published:** 2015-03-20

**Authors:** Kristoff Muylle, Patrick Flamen, Danielle J. Vugts, Thomas Guiot, Ghanem Ghanem, Nathalie Meuleman, Pierre Bourgeois, Bruno Vanderlinden, Guus A. M. S. van Dongen, Hendrik Everaert, Mélanie Vaes, Dominique Bron

**Affiliations:** 1 Jules Bordet Institute, Université Libre de Bruxelles, Brussels, Belgium; 2 VU University Medical Centre, Amsterdam, The Netherlands; 3 UZ Brussel, Vrije Universiteit Brussel, Brussels, Belgium; 4 MIMA Research Group, Vrije Universiteit Brussel, Brussels, Belgium; 5 Department of Nuclear Medicine, Jules Bordet Institute, Université Libre de Bruxelles, Waterloolaan 121, B-1000 Brussels, Belgium

**Keywords:** Radioimmunotherapy, CD20+ B-cell lymphoma, ^90^Y-Rituximab, Rituximab preload, Immuno-PET, ^89^Zr-rituximab, Dosimetry

## Abstract

**Purpose:**

To compare using immuno-PET/CT the distribution of ^89^Zr-labelled rituximab without and with a preload of unlabelled rituximab to assess the impact of preloading with unlabelled rituximab on tumour targeting and radiation dose of subsequent radioimmunotherapy with ^90^Y-labelled rituximab in CD20+ B-cell lymphoma.

**Methods:**

Five patients with CD20+ B-cell lymphoma and progressive disease were prospectively enrolled. All patients underwent three study phases: initial dosimetric phase with baseline ^89^Zr-rituximab PET/CT imaging without a cold preload, followed 3 weeks later by a second dosimetric phase with administration of a standard preload (250 mg/m^2^) of unlabelled rituximab followed by injection of ^89^Zr-rituximab, and a therapeutic phase 1 week later with administration of unlabelled rituximab followed by ^90^Y-rituximab. PET/CT imaging and tracer uptake by organs and lesions were assessed.

**Results:**

With a cold rituximab preload, the calculated whole-body dose of ^90^Y-rituximab was similar (mean 0.87 mSv/MBq, range 0.82–0.99 mSv/MBq) in all patients. Without a preload, an increase in whole-body dose of 59 % and 87 % was noted in two patients with preserved circulating CD20+ B cells. This increase in radiation dose was primarily due to a 12.4-fold to 15-fold higher dose to the spleen without a preload. No significant change in whole-body dose was noted in the three other patients with B-cell depletion. Without a preload, consistently higher tumour uptake was noticed in patients with B-cell depletion.

**Conclusion:**

Administration of the standard preload of unlabelled rituximab impairs radioconjugate tumour targeting in the majority of patients eligible for radioimmunotherapy, that is patients previously treated with rituximab-containing therapeutic regimens. This common practice may need to be reconsidered and further evaluated as the rationale for this high preload has its origin in the “prerituximab era”.

Clinical Trial Application: CTA 2011-005474-38

Trial Registry: EudraCT

**Electronic supplementary material:**

The online version of this article (doi:10.1007/s00259-015-3025-6) contains supplementary material, which is available to authorized users.

## Introduction

Radioimmunotherapy (RIT) is the targeting of a monoclonal antibody (mAb) coupled to a radioisotope to selectively deliver ionizing radiation to tumours [1]. As lymphoma cells are inherently radiosensitive, the CD20 antigen provides an excellent target for RIT because it is expressed at a high surface density in most lymphomas [2]. Following RIT, both malignant and normal B cells are depleted, with normal B cells recovering within 6 months [3].

The most widely studied radioconjugates for the treatment of B-cell non-Hodgkin’s lymphoma (NHL) are murine anti-CD20 mAbs radiolabelled with ^131^I (tositumomab, Bexxar®; GlaxoSmithKline, Brentford, UK; no longer available) or with the pure β-emitting isotope ^90^Y (ibritumomab tiuxetan, Zevalin®; Spectrum Pharmaceuticals Inc., Henderson, NV). In Europe, only ^90^Y-ibritumomab has been licensed, and it is used in combination with a preload of unlabelled rituximab [4]. Several studies have shown the efficacy of RIT in patients with CD20+)B-cell NHL, both as a single agent in indolent lymphoma and in combination with chemotherapy in indolent and aggressive lymphoma [3, 5–9]. Recently, the feasibility of RIT with ^90^Y-rituximab using a ^90^Y-ibritumomab treatment schedule has been reported [10].

As normal tissue toxicity (particularly myelosuppression) is dose limiting for RIT, the therapeutic index for RIT is thought to be enhanced by the use of excess unlabelled (“cold”) antibodies before RIT [2]. Preloading with unlabelled antibodies is thought to prevent normal tissue toxicity by providing a more predictable biodistribution profile of radiolabelled antibodies, decreasing clearance rates and prolonging the circulating half-life of the radiolabelled antibody [1, 11–13]. This preload is assumed to clear the peripheral blood of B cells and enhance targeting of the radiolabelled antibody to tumour cells. Despite the common use of a preload of unlabelled antibodies before RIT [14, 15], including its inclusion in clinical guidelines [4], little is known about the potential impact of high levels of circulating anti-CD20 antibodies on the targeting of a subsequent radiolabelled anti-CD20 antibody.

The further refinement of RIT has evolved to include consideration of the use of immuno-PET technology in its application [16]. Immuno-PET, the combination of PET and a radiolabelled mAb, combines the high sensitivity and resolution of a PET camera with the specificity of a mAb [17, 18]. PET is better suited than SPECT to tracer quantification [17], while targeting information can be combined with anatomical information when PET/CT is used [19]. Apart from its diagnostic capabilities and use in treatment planning, immuno-PET has potential for quantification of molecular interactions, which is particularly attractive when it is used for simulation of subsequent antibody-based therapy.

The majority of available PET isotopes are not appropriate for routine PET imaging because of unsuitable half-lives, poor availability, high production costs, and poorly developed radiochemistry [18]. ^89^Zr, which is a transition metal in group IVB of the periodic table, decays by positron emission (fraction 23 %) and electron capture (77 %) to ^89^Y. The concomitant gamma decay of ^89^Zr of 908.97 keV has no significant influence on the quantitative accuracy of PET images because these high-energy photons, with an energy far exceeding 511 keV, can be easily circumvented by the energy window of the PET scanner. ^89^Zr has a half-life of 78.4 h, which is compatible with the time needed for a mAb to achieve optimal tumour-to-background ratios. ^89^Zr can be obtained in high yield and radionuclide purity, and with low production costs. Moreover, ^89^Zr has ideal characteristics for optimal image quality and accurate quantification.

In what we be believe to be the first report of the use of ^89^Zr-rituximab, the aim of this study was to compare the distribution of ^89^Zr-rituximab with and without a standard preload of unlabelled rituximab in patients with relapsed CD20+ B-cell lymphoma, with the aim of assessing the potential impact of circulating anti-CD20 antibodies on whole-body distribution, radiation dose and tumour targeting of a subsequent radiolabelled anti-CD20 antibody as part of a RIT regimen.

## Materials and methods

### Radioconjugate production

^89^Zr-Rituximab was produced in a Good Manufacturing Practice facility with a manufacturing license at the VU/VUmc campus (Amsterdam, Netherlands). ^89^Zr (2.7 GBq/mL in 1 M oxalic acid) was produced by the BV cyclotron using a (p,n) reaction on natural ^89^Y and isolated using a hydroxamate column. The starting point of the chelator was desferrioxamine B (Desferal®; Novartis Pharma Stein AG, Stein, Switzerland), which was converted to its succinylated form, *N*-succinyldesferrioxamine B (N-sucDf). The hydroxamate groups of N-sucDf are temporarily blocked with iron [Fe(III)], and the N-sucDf-Fe form was esterified with 2,3,5,6-tetrafluorophenol (TFP) to the bifunctional chelator, TFP-N-sucDf-Fe. Rituximab (Mabthera®; Roche, Basel, Switzerland) was labelled with ^89^Zr starting from the chelate, TFP-N-sucDf-Fe, as described previously [20]. In brief, rituximab was modified by coupling of a Fe-N-sucDf-TFP ester at room temperature (pH 9.5–9.7). The iron was then removed from the chelate with an excess of ethylenediaminetetraacetic acid at 35 °C (pH 4.2–4.5), and the modified protein was purified on a pyrogen-free PD-10 column. The modified rituximab was labelled with ^89^Zr by first neutralizing the pH of the ^89^Zr solution with sodium carbonate and the protein was added together with 4-(2-hydroxyethyl)-1-piperazineethanesulphonic acid buffer. After 60 min, the ^89^Zr-labelled protein was purified on a pyrogen-free PD-10 column, and the product filtered. The immunoreactivity of ^89^Zr-rituximab was evaluated using a Lindmo cell binding assay [21], using 0.1 % paraformaldehyde-fixed NHL RAMOS cells, with an immunoreactive fraction above 70 % for each individual labelling procedure.

### Study population

Five patients with CD20+ B-cell lymphoma and progressive disease, and at least one prior treatment regimen were enrolled. Treatment with rituximab had to be stopped at least 6 months before inclusion.

### Methodology

The study comprised three phases:

1. Diagnostic/dosimetric phase I: Baseline ^89^Zr-rituximab dynamic PET/CT imaging after injection of ^89^Zr-rituximab (111 MBq) without a preload of unlabelled rituximab.
2. Diagnostic/dosimetric phase II (3 weeks later): Administration of a standard preload (250 mg/m^2^) of unlabelled rituximab followed 1 to 3 h later by injection of ^89^Zr-rituximab and dynamic PET/CT imaging.
3. Therapeutic phase (1 week later): Administration of 250 mg/m^2^ of unlabelled rituximab followed by slow intravenous injection of ^90^Y-rituximab, using the same doses as recommended for treatment with ^90^Y-ibritumomab tiuxetan (Zevalin®; 0.3 mCi/kg, 11.1 MBq/kg, if platelet count 100,000 to ≤150,000/mm^3^, 100 to ≤150 × 10^9^/L; and 0.4 mCi/kg, 14.8 MBq/kg, if platelet count >150,000/mm^3^, >150 × 10^9^/L) [4].

### PET/CT imaging

PET/CT scans were performed with a dedicated BGO PET system coupled to a helical CT scanner (Discovery LS; GE Healthcare Technologies, Milwaukee, WI), and PET/CT images were visualized on a dedicated viewing station (Advantage Windows 4.5; GE).

#### PET with ^89^Zr-rituximab

Whole-body PET scans consisting of seven or eight bed positions covering the patient from the base of the skull to the upper thighs were obtained. At each bed position, a 5-min emission scan in three-dimensional mode was acquired. Whole-body scans were completed at three time-points starting within 1 h and at 72 h and 144 h after the first intravenous injection of 111 MBq ^89^Zr-rituximab. All scans were normalized and corrected for randoms, scatter, attenuation and decay. The images (matrix 128 × 128) were reconstructed using an attenuation and a normalization-weighted ordered subsets expectation maximization (OSEM) algorithm (Advance 6.0), with five iterations and 32 subsets followed by postsmoothing of the reconstructed images using an 8-mm full-width at half-maximum Gaussian filter.

#### CT scan

CT was performed in all patient studies at “low dose”, without administration of oral or intravenous contrast agent. The tube current intensity used by the CT scanner was determined by Auto-mA®, a dose-reduction algorithm provided by the camera vendor that modulates the tube current intensity during acquisition depending on a noise index and the attenuation information given by the planar scout image. The noise index for low-dose CT scans was 28. The current intensity ranged from 30 to 200 mA, and the voltage was set to 120 kV. The whole-body absorbed dose for the CT scans ranged between 3 and 5 mSv. The CT map was used for attenuation correction of the PET scans and as a structural correlate to optimize PET image interpretation.

### Contouring

Organs were contoured on the anatomical images obtained from the CT scans using the DosiSoft® station. PET/CT coregistration was checked visually. The contours were then projected on the PET images for statistical analysis. The considered organs (for which residence times were calculated) were the liver, spleen, bone marrow (skeleton), kidneys, lungs, gonads and thyroid. The activity for calculating the residence times for the remainder of the body was obtained from whole-body (head to thigh) volumes of interest (VOIs), minus the sum of the activities of the considered organs. The bone marrow activity was assessed by automatic segmentation of the skeleton (excluding the skull) performed on CT images using OWS® 1.0.

Several contouring methods were applied for the lesions due to the great variability in visibility, both on CT and PET images. When lesions were identifiable on PET images (i.e. with an activity concentration higher than background activity), lesions were segmented with a fixed threshold of 42 % of the maximum activity value. When there was an overlap between lesions, the CT images helped discriminate the different lesions. When lesions were not identifiable on PET or automated segmentation was not reliable (i.e. because the contrast was too low or because of proximity to organs with high uptake), two options were possible: (1) if the lesion was clearly identifiable on CT images, contours were drawn on the CT images and then projected on the PET images; and (2) if the lesion was not clearly identifiable on CT images, contours were drawn on the day-6 PET images (where lesion contrast was often the highest) using the method described above, and earlier PET images were then registered on the day-6 images and the contours were projected, and manual corrections were applied if registration was imperfect.

### Quantification

The contours were exported in RT Structure format, then imported into PMOD (PMOD Technologies Ltd, Zürich, Switzerland) for quantification. The total activity (average activity in a VOI multiplied by the volume) measured by PMOD was divided by the injected activity (with decay correction) resulting in the uptake percentage of organs and lesions.

### Dosimetry

Dosimetry was performed using OLINDA/EXM®. For ^89^Zr dosimetry, the time–activity curve (TAC) was plotted using the activity values given by PMOD. For ^90^Y dosimetry, the activity values were calculated from the ^89^Zr activities according to the equation:

$$ {A}_{90\mathrm{Y}}\left({t}_{\mathrm{acq}}\right)={A}_{89\mathrm{Z}\mathrm{r}}\left({t}_{\mathrm{acq}}\right)\times {e}^{\ln \kern0.1em (2)\times \left({t}_{\mathrm{acq}}-{t}_{\mathrm{inj}}\right)\times \left(\frac{1}{t_{89\mathrm{Z}\mathrm{r}}}-\frac{1}{t_{90\mathrm{Y}}}\right)} $$

Where *A*_90Y_(*t*_acq_) and *A*_89Zr_(*t*_acq_) are the activities calculated and measured, respectively, at the different acquisitions times, *t*_inj_ is the time of injection and *t*_89Zr_ and *t*_90Y_ are the half-lives of the isotopes.

The total area under the TAC was calculated by summing the following areas, with the following approximations: (1) the area for the interval between time zero (injection) and time 1 (1 h after injection) calculated using a rectangle; (2) the area between the three acquisition time-points calculated using trapezoids between each time-point; and (3) the area after the third time-point calculated using the physical decay. The total area under the curve was then divided by the injected activity (at time zero) to give the time-integrated activity coefficient, which was inputted into OLINDA/EXM® (residence times for each considered organ are available in the Supplementary Online Table 1).

.

## Results

Patient characteristics are shown in Table 1. Five patients with CD20+ B-cell lymphoma with progressive disease and who had at least one prior treatment regimen were enrolled. Four patients were treated for a relapse of follicular lymphoma and one for a nodular lymphocyte-predominant, CD20+ Hodgkin’s lymphoma. None of the patients had bone marrow involvement. The median age of the patients was 51 years (range 41–62 years) and all were male. The median number of previous treatment lines was three (range one to four). The two patients with fewer than three previous treatments (patients 1 and 2) had preserved circulating CD20+ lymphocytes (assessed by immunophenotyping in the blood before inclusion in this study), while the other three patients had B-cell depletion (patients 3, 4 and 5) due to three or more prior treatment lines (although immunophenotyping was not available for patient 4, this patient was considered to have B-cell depletion having been previously treated with autologous stem cell transplantation and RIT). A detailed overview of the effective and absorbed doses for ^89^Zr-rituximab and ^90^Y-rituximab is available in the Supplementary Online Tables 2–5.

**Table 1:** Patient characteristics

Patient no.	Sex	Age (years)	Histology	Disease stage at study entry	No. of prior therapies	Time to last rituximab dose at study entry (months)	Total lymphocytes (/mm^3^)	Total lymphocytes (%)	CD20+ lymphocytes (/mm^3^)	CD20+ lymphocytes (%)	Haematological toxicity after ^90^Y-rituximab (CTCAE v.3)^a^	Response after ^90^Y-rituximab
1	Male	41	NLPHL	I Bulky	1	24	2020	31	222	9	2	Complete remission
2	Male	51	Follicular lymphoma	IV	2	18	860	30	72	5	3	Complete remission
3	Male	55	Follicular lymphoma	II Bulky	3	8	710	10	0	0	1	Partial remission
4	Male	46	Follicular lymphoma	II	3	15	1070	15	Not available	Not available	1	Complete remission
5	Male	61	Follicular lymphoma	II	4	6	940	35	0	0	2	Complete remission

*NLPHL* nodular lymphocyte-predominant Hodgkin’s lymphoma

^a^Haematological toxicity was transient

**Fig. 1:**
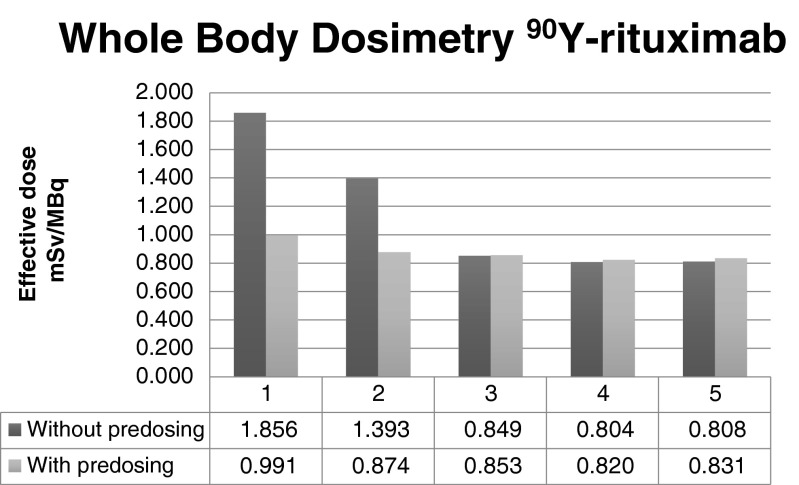
Whole-body dosimetry of ^90^Y-rituximab with and without a preload of unlabelled (“cold”) rituximab antibodies

With a preload of unlabelled rituximab, the calculated effective (whole-body) doses for ^90^Y-rituximab were similar in all patients (mean 0.87 mSv/MBq, range 0.82–0.99 mSv/MBq). Without a preload, the whole-body radiation doses were 59 % and 87 % higher than with a preload in patients 1 and 2, respectively, but were not significantly different in the three other patients (Fig. 1).

**Fig. 2:**
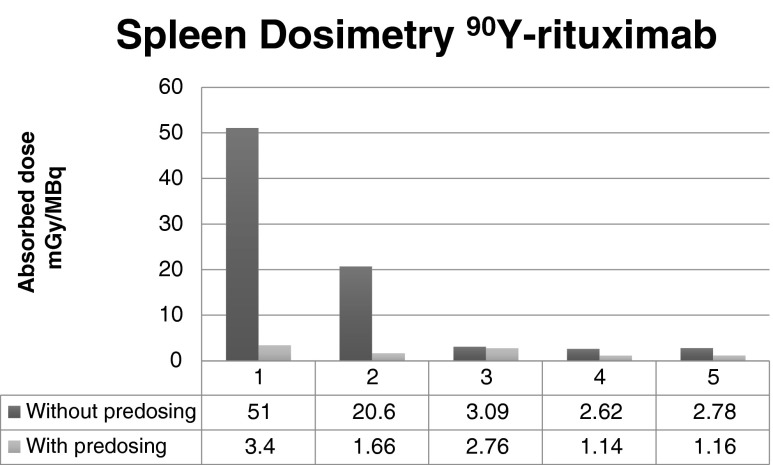
Radiation dose to the spleen as a function of the amount of circulating CD20+ lymphocytes

**Fig. 3:**
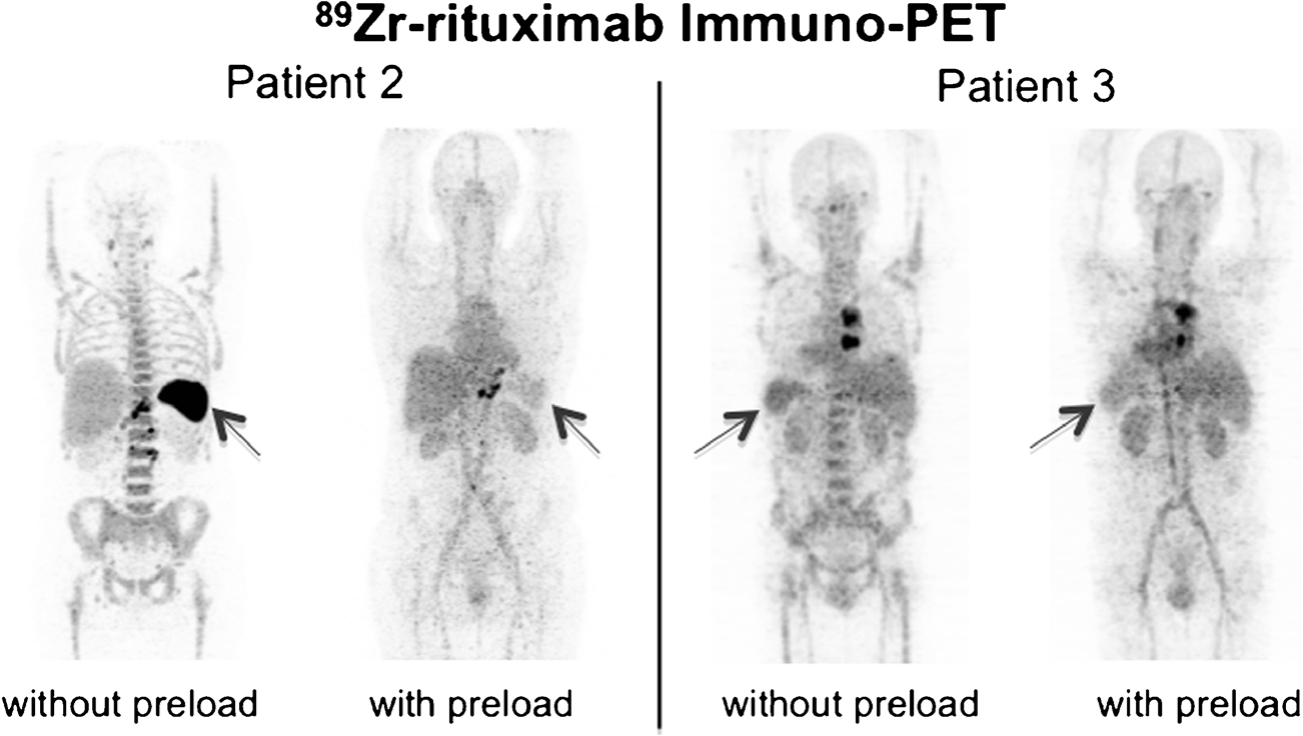
^89^Zr-rituximab immuno-PET images obtained 6 days after injection in a patient (patient 2; anterior view) with a preserved amount of circulating CD20+ lymphocytes and a patient (patient 3; posterior view) with B-cell depletion

The higher whole-body radiation doses without a preload in patients 1 and 2 were primarily due to higher radiation doses to the spleen (Fig. 2). Without a preload of rituximab, the uptake of the radioconjugate, ^90^Y-rituximab, was 12.4-fold to 15-fold higher in these two patients and only 1.1-fold to 2.4-fold higher in the other three patients. Correlation with the number of previous treatment lines in each patient showed that the higher tracer uptake in the spleen, and hence the higher whole-body radiation dose, was much higher in the two patients who had had only one or two previous treatment regimens, and was only moderately higher in the three patients who had three or four previous treatment regimens. Correlation of the percentage of circulating B cells revealed that the influence of a preload of rituximab on the distribution of the radioconjugate, especially uptake in the spleen, depended highly on the amount of uptake of circulating CD20+ lymphocytes in the spleen (Fig. 3). A major influence of the preload is noted in the two patients with preserved (5–9 %) circulating CD20+ lymphocytes, while only a minor influence on the radiation dose to the spleen was seen in patients with B-cell depletion (0 % circulating CD20+ lymphocytes).

**Fig. 4:**
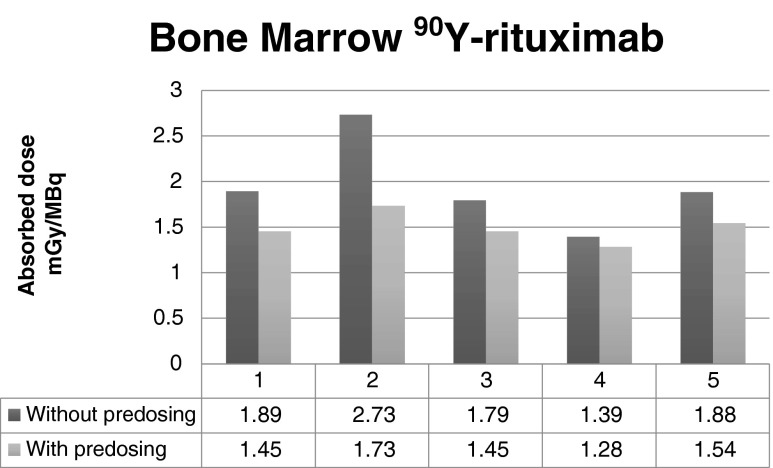
Influence of a preload of unlabelled rituximab on the radiation dose to the bone marrow

**Fig. 5:**
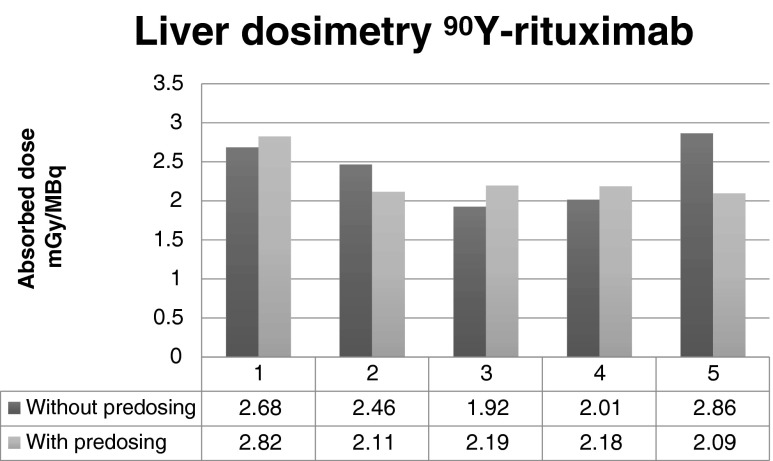
Influence of a preload of unlabelled rituximab on the radiation dose to the liver

Without a preload, the radiation doses to the bone marrow were 9 % to 58 % higher than with a preload (Fig. 4), while the radiation doses to the liver were similar with and without a preload (Fig. 5).

For ^89^Zr-rituximab, the calculated effective (whole-body) doses after a preload of unlabelled rituximab were similar in all patients (mean 0.41 mSv/MBq, range 0.39–0.44 mSv/MBq). Without a preload, the whole-body radiation doses were 37 % and 55 % higher than with a preload in the two patients with preserved circulating CD20+ lymphocytes, primarily due to higher radiation doses to the spleen, but were not significantly different in the three patients with B-cell depletion (Table 2).

**Table 2:** Absorbed doses to the liver, spleen and red marrow, and effective whole-body dose for ^89^Zr-rituximab

	Without preload						With preload					
	Patient 1	Patient 2	Patient 3	Patient 4	Patient 5	Mean ± SD	Patient 1	Patient 2	Patient 3	Patient 4	Patient 5	Mean ± SD
Liver (mGy/MBq)	1.13	1.08	0.86	0.89	1.21	1.03 ± 0.15	1.22	0.95	0.97	0.95	0.94	1.01 ± 0.12
Red marrow (mGy/MBq)	0.59	0.79	0.59	0.50	0.62	0.62 ± 0.11	0.54	0.58	0.53	0.48	0.54	0.53 ± 0.03
Spleen (mGy/MBq)	12.30	5.35	0.98	0.89	0.92	4.09 ± 4.97	1.10	0.65	0.93	0.53	0.53	0.75 ± 0.26
Whole body (mSv/MBq)	0.68	0.57	0.41	0.40	0.43	0.50 ± 0.12	0.44	0.42	0.42	0.39	0.40	0.41 ± 0.02

**Fig. 6:**
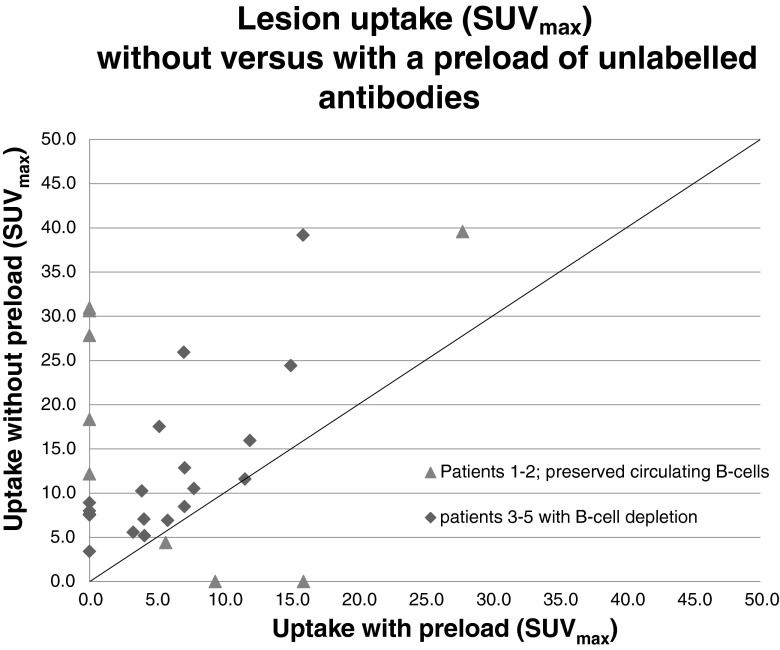
Comparison of lesion uptake (SUV_max_) without and with a preload of unlabelled rituximab

**Fig. 7:**
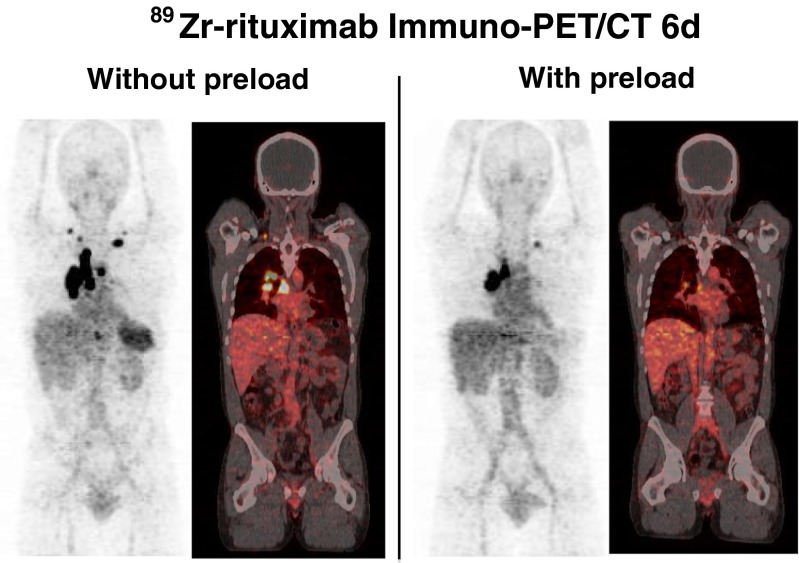
^89^Zr-rituximab immuno-PET/CT images in patient 4 (with CD20+ B-cell depletion) obtained 6 days after injection without and with a preload of unlabelled rituximab show consistently better tumour targeting without the preload

**Fig. 8:**
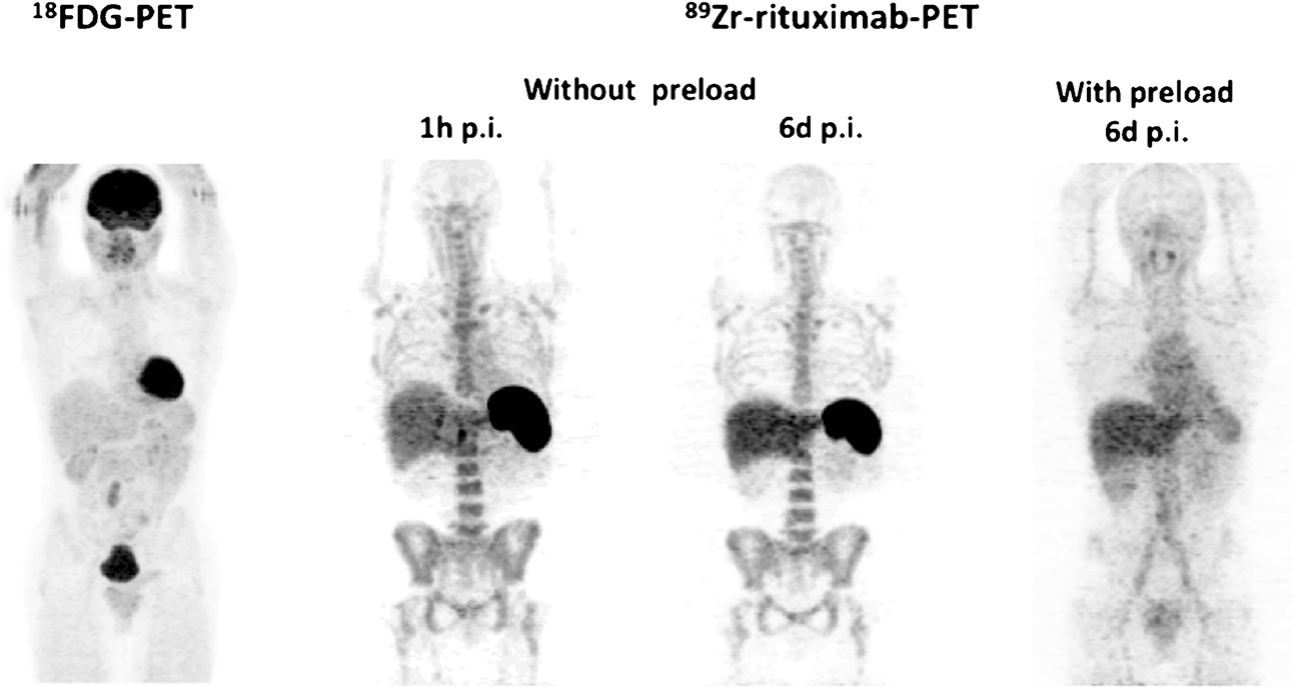
^18^F-FDG PET and ^89^Zr-rituximab immuno-PET images in patient 1 obtained 1 h after injection and 6 days after injection without a preload of unlabelled rituximab and 6 days after injection with a preload of unlabelled rituximab show higher uptake in the involved lymph node with the preload as a result of a higher residence time of the tracer in the blood circulation

**Fig. 9:**
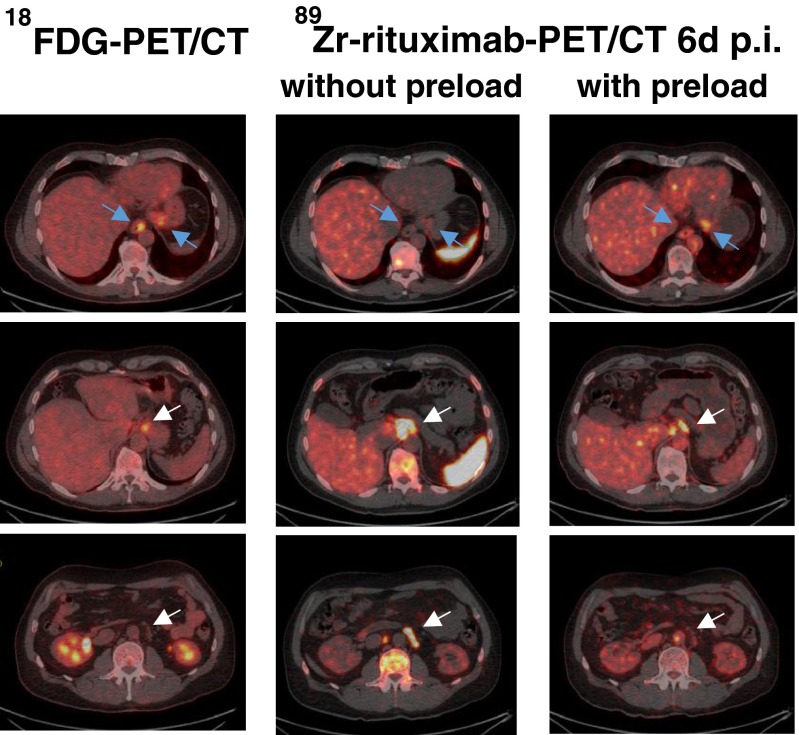
^18^F-FDG PET/CT and ^89^Zr-rituximab immuno-PET/CT images in patient 2 obtained 6 days after injection with and without a preload of unlabelled rituximab show lower tracer uptake in involved lymph nodes with the preload (*white arrows*), but higher uptake in less accessible visceral lesions (oesophagus and stomach; *blue arrows*) resulting in better tumour targeting

**Fig. 10:**
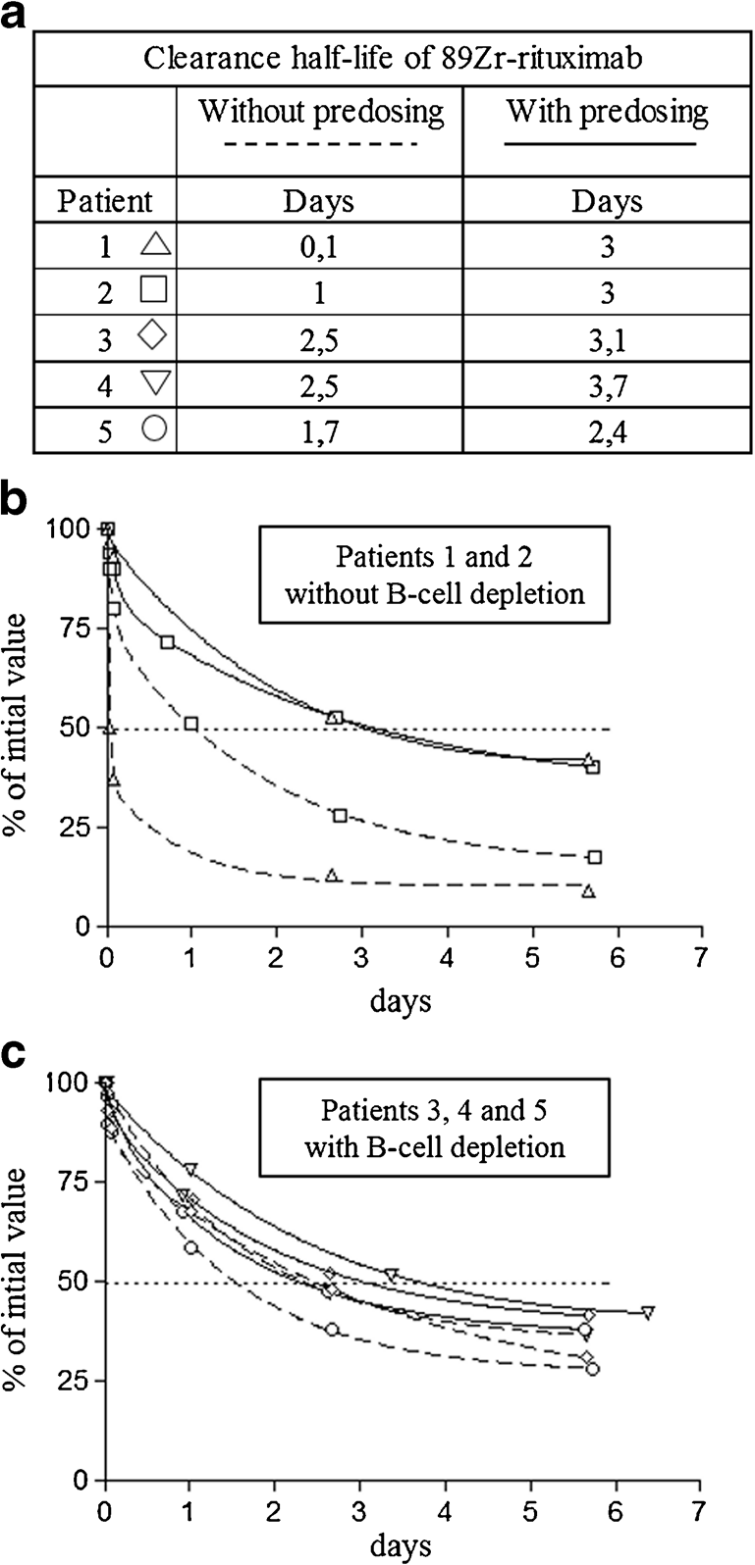
Pharmacokinetics. After intravenous administration of 111 MBq ^89^Zr-rituximab, blood samples were obtained from the arm opposite the infusion side 10 min, 1 and 2 h, and then 1, 3 and 6 days after infusion. At each time-point, ^89^Zr disintegration counts/unit volume were measured using a calibrated γ-well counter corrected for ^89^Zr physical decay. **a** Clearance half-life of ^89^Zr-rituximab with and without predosing. **b**, **c** Blood concentration of ^89^Zr-rituximab expressed as percentages of the initial 10-min value in (**b**) patients 1 and 2 with preserved circulating CD20+ lymphocytes and (**c**) patients 3, 4 and 5 with B-cell depletion

Comparison of the maximum standardized uptake value (SUV_max_) of the 30 lesions in the five patients (Fig. 6) using the ^89^Zr-rituximab immuno-PET/CT images taken 6 days after injection showed a consistently higher lesion uptake (tumour targeting) without a preload in all three patients with B-cell depletion (as illustrated in patient 4 in Fig. 7). In the two patients with preserved circulating CD20+ lymphocytes, three lesions showed less or no uptake without a preload, while other lesions showed higher uptake. A more detailed evaluation of these three lesions with low uptake revealed the reasons for this finding. In patient 1 (with 9 % circulating CD20+ lymphocytes) with the preload, uptake in an involved lymph node was higher as a result of lower uptake in the spleen leading to a higher residence time of the tracer in the blood circulation. Images obtained without the preload showed intense uptake in the spleen and fast clearance of the injected tracer from the blood 1 h after ^89^Zr-rituximab injection (Fig. 8). In patient 2 (with 5 % circulating CD20+ lymphocytes) with the preload, tracer uptake in involved lymph nodes was lower on the one hand, but on the other hand was higher in the two visceral lesions as a result of lower uptake in the spleen leading to a higher residence time of the tracer in the blood circulation and binding of the radioconjugate in less accessible regions (Fig. 9). The pharmacokinetics of ^89^Zr-rituximab confirmed the fast clearance of ^89^Zr-rituximab from the blood in both patients (particularly patient 1) with preserved circulating CD20+ lymphocytes without preload and the significantly slower clearance of the radioconjugate (comparable with the clearance in patients with B-cell depletion) with a preload (Fig. 10).

## Discussion

The efficacy of RIT in patients with B-cell NHL as a single agent in indolent lymphoma and in combination with chemotherapy in both indolent and aggressive lymphoma has been reported [3, 5–9]. The anti-CD20 antibodies, ^90^Y-ibritumomab tiuxetan and ^131^I-tositumomab, are the only radiolabelled antibodies licensed for RIT, with ^90^Y-ibritumomab tiuxetan being currently the only commercially available agent.

There is interest in developing a RIT regimen incorporating radiolabelled rituximab, which is part of the standard of care in NHL [22–25]. Rituximab is a chimeric IgG1 kappa mAb targeting the same epitope on the CD20 antigen as the murine mAb ibritumomab, and which is predicted to increase immune-based antitumour activity, improve pharmacokinetics and reduce immunogenicity compared to currently available radioconjugates. RIT with ^90^Y-rituximab in CD20+ B-cell lymphoma has shown promising efficacy and tolerability when utilizing the ^90^Y-ibritumomab tiuxetan schedule [10].

The efficacy of RIT is dependent on the properties of the targeted antigen (i.e. specificity, tumour selectivity, density, availability, shedding, heterogeneity of expression), the properties of the tumour (i.e. vascularization, blood flow, permeability), the properties of the antibody, and the properties of the radioisotope (i.e. emission characteristics, half-life, bioavailability) [14, 26]. The therapeutic index of RIT is thought to be improved if it is preceded by administration of excess cold antibodies that prolongs the circulating half-life of the radiolabelled antibody and blocks nonspecific binding to normal tissues resulting in increased tumour retention of the radiolabelled antibody [12, 14].

Despite the common use of a preload of cold antibodies before RIT [4], little is known about the potential impact of such high levels of circulating anti-CD20 antibodies on tumour targeting of a subsequently administered radiolabelled anti-CD20 antibody. This common practice is based on a small phase 1/2 dosimetry study using ^111^In-labelled ibritumomab tiuxetan planar imaging in six patients [27]. In three of these patients, a preload of 100 mg/m^2^ rituximab was administered, while the other three patients received 250 mg/m^2^. The 250 mg/m^2^ rituximab dose was chosen as the dose to be given before RIT because no difference in imaging or dosimetry was observed between dosing groups, and there was potential for enhanced clinical response from the higher dose of rituximab. However, no comparison has been made on the distribution without a predose of unlabelled antibodies. Furthermore, the study evaluated the impact of a preload with chimeric antibodies (rituximab) on targeting of a second radiolabelled murine anti-CD20 antibody (ibritumomab).

To the best of our knowledge, our study is the first to compare the distribution of ^89^Zr-rituximab with and without a standard preload of unlabelled rituximab in patients with relapsed CD20+ B-cell lymphoma. Although this study was conducted in a small group of patients, striking differences in the influence of the standard preload dose of unlabelled rituximab (250 mg/m^2^) between patients with and without B-cell depletion were observed. In patients with B-cell depletion, which represents the majority of patients currently treated with RIT, the preload of unlabelled rituximab had no significant influence on whole-body radiation dose, yet consistently impaired tumour targeting due to a partial saturation of CD20 receptors present on lymphoma cells. In contrast, in the two patients with preserved circulating CD20+ lymphocytes, the preload of unlabelled rituximab cleared circulating B lymphocytes from the blood, reduced whole-body radiation dose, significantly reduced radioactivity uptake in the spleen, and resulted in slower clearance of the radioconjugate from the circulation.

The preload of cold rituximab consistently impaired tumour targeting in the three patients with B-cell depletion. In contrast, it had a variable influence on tumour targeting in the two patients with preserved circulating CD20+ lymphocytes, enhancing uptake in three lesions (of which two were visceral) by improving biodistribution and preventing sequestration of the radioconjugate by the antigen sink, while impairing targeting of other tumour sites due to partial saturation with unlabelled rituximab.

Although clinical studies have shown that high and even multiple doses of induction therapy with rituximab alone or as part of rituximab-containing chemotherapy regimens do not appear to compromise the clinical efficacy of subsequent anti-CD20-based RIT [28–30], and that high serum levels of rituximab significantly increase the effective half-life of subsequent radiolabelled rituximab [29], the key issue of the optimal treatment approach for RIT remains to be elucidated. New treatment approaches, such as fractionated RIT [29, 31] or dual-targeted antibody/radioantibody therapy [32], have the potential to further improve the clinical efficacy of RIT. A recent phase I study [33] has shown encouraging results in patients with relapsed/refractory aggressive lymphoma using a fractionated dosing schedule along with the dual-targeting approach with the intention of improving the delivery and retention of the radioconjugate at the tumour sites. In that study, unlabelled anti-CD20 veltuzumab was administered to deplete the circulating B cells, enhancing biodistribution of the anti-CD22 radioconjugate ^90^Y-epratuzumab tetraxetan without interfering with tumour targeting.

The inherent heterogeneity of radiopharmaceutical distribution in target lesions and normal organs suggests that a individualized patient-tailored approach might be of additional value in RIT. The antigen sink, which depends on several parameters such as tumour burden, spleen volume and amount of circulating B-lymphocytes, plays a key role in the amount of rituximab needed to ensure adequate serum levels (effective half-life) of the radioconjugate. Other factors influencing tumour targeting by the radioconjugate are tumour heterogeneity and site of tumour involvement. Adequate receptor imaging might be a promising tool for the evaluation of the influence of these multiple factors on tumour targeting, for imaging-based three-dimensional calculation of the absorbed dose for organs and lesions, and for simulation of different treatment approaches. In the five patients in this study a preload of rituximab improved the biodistribution of the radioconjugate by preventing its sequestration in patients with preserved circulating CD20+ lymphocytes, but consistently impaired radioconjugate tumour targeting in patients with B-cell depletion, the latter representing the majority of patients eligible for RIT in the “rituximab era”. However, the small number of patients in this study along with the multiple parameters influencing tumour targeting in RIT in CD20+ lymphoma do not allow drawing definitive conclusions concerning the optimal treatment approach in RIT to be drawn, and this should be the basis of further studies.

The advantage of using a positron-emitting isotope is that PET is inherently quantitative, whereas quantification with planar imaging involves considerable uncertainties that can often be greater than estimated activity concentrations. ^89^Zr-rituximab PET/CT, as assessed in our study, provides an excellent imaging tool for accurate quantification of CD20 antigen expression, which is of particular interest for dosimetry as a prelude to RIT with ^90^Y-rituximab, allowing the possibility for dose–response correlation, prediction of treatment outcome, better selection of patients for receptor-targeted therapy, and patient-tailored image-guided therapy [17, 34–36].

The effective patient dose of ^89^Zr-rituximab (after a preload of unlabelled rituximab) was relatively high (0.41 mSv/MBq), probably due to the long half-life of the tracer and concomitant gamma decay of ^89^Zr of 908.97 keV. In this study we administered 111 MBq ^89^Zr-rituximab that resulted in an average total effective dose of 45.5 mSv, which is justifiable within the context of subsequent RIT with administration of 14.8 MBq/kg of ^90^Y-rituximab (1,184 MBq for a body weight of 80 kg), resulting in an average total effective dose of 1.03 Sv for RIT. Nevertheless, given the high image quality with 111 MBq in this study and the high sensitivity of new-generation (time-of-flight) PET scanners, lowering the injected activities to 74 MBq (or less by increasing the acquisition time, especially on day 6 after injection) while preserving the largely sufficient quantitative image accuracy should be feasible.

As there are currently no dosimetry data available for ^90^Y-rituximab, the doses were compared with those of ^90^Y-ibritumomab (the murine counterpart, binding the same epitope) [34], both after a preload of rituximab and using ^89^Zr-immuno-PET. The calculated mean effective dose was the same for both studies (0.87 mSv/MBq). Compared with the current study, the mean absorbed doses of ^90^Y-ibritumomab in the liver and spleen tend to be higher and those for kidneys and lungs somewhat lower (Table 3). The mean absorbed dose of ^90^Y-rituximab to the red marrow was threefold higher. Although some differences in pharmacokinetics between the two mAbs may be expected, it is more likely that the discrepancy in the absorbed doses are due to differences in methodology between studies. The major difference in methodology is for the assessment of the dose to the red marrow. In the current study, a novel three-dimensional image-based approach was used for assessing the bone marrow dose, while for the ^89^Zr-/^90^Y-ibritumomab study, red marrow dosimetry was based on blood samples, where the residence time was estimated assuming a red marrow radioactivity concentration of 30 % of the whole-blood activity concentration [37]. The latter approach may underestimate the red marrow dose if there is any interaction of the targeting agent with the bone marrow.

**Table 3:** Comparison of doses of ^90^Y-rituximab and ^90^Y-ibritumomab (after a preload of rituximab) for selected organs using ^89^Zr-immuno-PET

Study	Radioconjugate	Absorbed organ doses (mGy/MBq, mean ± SD)					Effective whole-body dose (mSv/MBq, mean ± SD)
		Liver	Spleen	Kidneys	Lungs	Red marrow	
Current study	^90^Y-Rituximab	2.28 ± 0.31 (2.09–2.82)	2.02 ± 1.01 (1.14–3.40)	2.24 ± 0.30 (1.75–2.54)	2.18 ± 0.16 (1.92–2.35)	1.49 ± 0.16 (1.28–1.73)	0.87 ± 0.07 (0.82–0.99)
[34]	^90^Y-Ibritumomab	3.2 ± 1.8 (1.5–6.6)	2.88 ± 0.67 (1.83–3.83)	1.46 ± 0.31 (0.99–1.88)	1.47 ± 0.34 (1.07–1.82)	0.52 ± 0.04 (0.48–0.58)	0.87 ± 0.14 (0.70–1.06)

The similar haematological toxicities (and efficacy) after ^90^Y-rituximab (using the Zevalin® treatment schedule) in the present study (Table 1) and a previous study [10] support the assumption that the threefold higher mean absorbed dose of ^90^Y-rituximab to the red marrow compared to that of ^90^Y-ibritumomab was more likely due to differences in methodology than to higher accumulation of the chimeric radioconjugate in the bone marrow. Another difference in methodology between the two studies is that the organ VOIs in the current study were drawn on the anatomical images obtained from the coregistered CT images (using PET/CT), while in the ^89^Zr-/^90^Y-ibritumomab study the images were acquired on a stand-alone PET scanner with the organs contoured on functional images. Finally, in the current study, whole-body VOIs (not including part of the legs) were used for calculating the residence times for the remainder of the body, that somewhat underestimated the effective whole body doses. However, this would not have affected the calculated absorbed organ doses of ^90^Y-rituximab, as the deposition of the energy of the radiation emitted by ^90^Y is limited to the organ only.

In conclusion, the results of our study show that the administration of the standard preload of unlabelled rituximab impairs tumour targeting of the radioconjugate in the majority of patients eligible for RIT, primarily patients with B-cell depletion due to prior treatment with rituximab-containing therapeutic regimens. As the rationale for this high predose has its origin in the “prerituximab era”, this common practice may need to be reconsidered and further evaluated, in particular in the different setting of the licensed radioconjugate ^90^Y-ibritumomab tiuxetan (Zevalin®), where a chimeric antibody (rituximab) is used as a preload for subsequent RIT with a second radiolabelled murine anti-CD20 antibody (ibritumomab). Our observations also suggest that imaging with a mAb labelled with a positron-emitting isotope, such as ^89^Zr, in combination with PET could be useful for visualizing the biodistribution of the individual radiotracer, and may have utility in the elucidation of the dose–response of RIT and in defining patients at high risk of toxicity. Further investigation is warranted to confirm the potential role of immuno-PET in receptor-targeted therapies.

## Electronic supplementary material

Below is the link to the electronic supplementary material.

ESM 1 (PDF 49.1 kb)

ESM 2 (PDF 51 kb)

ESM 3 (PDF 56 kb)

ESM 4 (PDF 51 kb)

ESM 5 (PDF 56 kb)
