# Supplementary material for: Tumour targeting and radiation dose of radioimmunotherapy with 90Y-rituximab in CD20+ B-cell lymphoma as predicted by 89Zr-rituximab immuno-PET: impact of preloading with unlabelled rituximab
Source: Eur J Nucl Med Mol Imaging. 2015 Mar 20;42(8):1304–14. doi: 10.1007/s00259-015-3025-6 (PMC4480335; doi:10.1007/s00259-015-3025-6)
Supplement: Supplementary file 1 — (PDF 49.1 kb) [file 259_2015_3025_MOESM1_ESM.pdf]

Supplementary Table 1 Residence times (hours) for 89Zr- & 90Y-rituximab

	**89Zr-rituximab**									
	Without predosing					With predosing				
	1	2	3	4	5	1	2	3	4	5
Skeleton	10,03	14,68	9,09	6,62	9,36	6,90	8,40	6,85	6,02	7,29
Liver	11,49	10,50	7,98	8,36	12,03	12,08	8,98	9,11	9,09	8,96
Spleen	20,44	8,64	1,24	1,11	1,11	1,41	0,68	1,13	0,47	0,47
Kidneys	0,76	0,78	1,45	1,54	1,48	1,54	1,19	1,56	1,76	1,64
Gonads	0,01	0,02	0,03	0,02	0,03	0,07	0,05	0,03	0,01	0,04
Lungs	1,86	4,41	4,54	4,43	3,10	4,69	5,03	4,86	4,82	4,09
Thyroid	0,01	0,01	0,01	0,03	0,02	0,03	0,01	0,01	0,03	0,02
Remainder	26,82	40,64	50,20	51,37	55,67	56,17	54,51	57,53	52,72	55,60
	**90Y-rituximab**									
	Without predosing					With predosing				
	1	2	3	4	5	1	2	3	4	5
Skeleton	8,41	12,07	6,82	5,53	7,78	5,74	7,07	7,76	5,00	6,17
Liver	9,50	8,72	1,22	7,14	10,14	10,00	7,48	1,30	7,73	7,41
Spleen	17,85	7,20	1,08	0,92	0,97	1,19	0,58	0,97	0,40	0,41
Kidneys	0,63	0,66	3,91	1,29	1,23	1,28	1,00	4,15	1,45	1,35
Gonads	0,01	0,01	0,02	0,02	0,03	0,06	0,04	0,02	0,01	0,03
Lungs	1,61	3,91	7,55	3,80	2,72	4,02	4,36	5,69	4,14	3,56
Thyroid	0,01	0,01	0,01	0,02	0,01	0,02	0,01	0,01	0,02	0,02
Remainder	22,89	34,81	42,35	43,13	47,28	47,26	45,99	48,52	44,19	46,54
