# Supplementary material for: Tumour targeting and radiation dose of radioimmunotherapy with 90Y-rituximab in CD20+ B-cell lymphoma as predicted by 89Zr-rituximab immuno-PET: impact of preloading with unlabelled rituximab
Source: Eur J Nucl Med Mol Imaging. 2015 Mar 20;42(8):1304–14. doi: 10.1007/s00259-015-3025-6 (PMC4480335; doi:10.1007/s00259-015-3025-6)
Supplement: Supplementary file 2 — (PDF 51 kb) [file 259_2015_3025_MOESM2_ESM.pdf]

	**Supplementary Table 2** Absorbed Dose [mGy/MBq] 89Zr-rituximab (organs)									
	Without predosing					With predosing				
	1	2	3	4	5	1	2	3	4	5
Adrenals	0,612	0,565	0,457	0,447	0,521	0,521	0,473	0,488	0,457	0,470
Brain	0,126	0,188	0,204	0,202	0,224	0,220	0,218	0,224	0,205	0,218
Breasts	0,178	0,226	0,227	0,226	0,244	0,252	0,243	0,250	0,232	0,238
Gallbladder Wall	0,506	0,523	0,468	0,474	0,584	0,585	0,505	0,521	0,496	0,507
Lower large intestine wall	0,233	0,313	0,316	0,307	0,347	0,336	0,333	0,340	0,311	0,332
Small Intestine	0,296	0,352	0,344	0,338	0,386	0,378	0,363	0,374	0,344	0,364
Stomach Wall	0,616	0,460	0,337	0,335	0,373	0,381	0,348	0,369	0,333	0,346
Upper large intestine wall	0,304	0,354	0,344	0,341	0,393	0,387	0,365	0,376	0,348	0,366
Heart Wall	0,346	0,390	0,366	0,363	0,400	0,414	0,391	0,401	0,374	0,381
Kidneys	0,882	0,727	0,793	0,815	0,849	0,870	0,713	0,850	0,887	0,857
Liver	1,130	1,080	0,857	0,885	1,210	1,220	0,949	0,965	0,951	0,944
Lungs	0,433	0,654	0,624	0,610	0,532	0,669	0,679	0,670	0,648	0,594
Muscle	0,233	0,268	0,263	0,261	0,290	0,291	0,279	0,288	0,266	0,278
Ovaries	0,238	0,324	0,330	0,322	0,365	0,354	0,348	0,356	0,326	0,348
Pancreas	1,020	0,694	0,440	0,435	0,496	0,507	0,446	0,475	0,428	0,441
Red Marrow	0,586	0,788	0,585	0,496	0,622	0,536	0,576	0,531	0,480	0,536
Osteogenic Cells	0,440	0,603	0,517	0,469	0,557	0,511	0,527	0,512	0,465	0,507
Skin	0,143	0,176	0,182	0,182	0,202	0,202	0,194	0,202	0,186	0,195
Spleen	12,300	5,350	0,977	0,894	0,917	1,100	0,653	0,929	0,528	0,530
Testes	0,123	0,176	0,222	0,198	0,243	0,328	0,275	0,234	0,186	0,257
Thymus	0,204	0,278	0,289	0,287	0,309	0,317	0,310	0,318	0,295	0,304
Thyroid	0,157	0,214	0,231	0,296	0,273	0,322	0,255	0,261	0,300	0,291
Urinary Bladder	0,178	0,252	0,281	0,281	0,311	0,309	0,301	0,313	0,286	0,304
Uterus	0,221	0,298	0,316	0,313	0,350	0,344	0,336	0,347	0,318	0,337
