# Supplementary material for: Tumour targeting and radiation dose of radioimmunotherapy with 90Y-rituximab in CD20+ B-cell lymphoma as predicted by 89Zr-rituximab immuno-PET: impact of preloading with unlabelled rituximab
Source: Eur J Nucl Med Mol Imaging. 2015 Mar 20;42(8):1304–14. doi: 10.1007/s00259-015-3025-6 (PMC4480335; doi:10.1007/s00259-015-3025-6)
Supplement: Supplementary file 3 — (PDF 56 kb) [file 259_2015_3025_MOESM3_ESM.pdf]

	**Supplementary Table 3** Effective Dose [mSv/MBq] 89Zr-rituximab (whole body)									
	Without predosing					With predosing				
	1	2	3	4	5	1	2	3	4	5
Adrenals	0,0015	0,0014	0,0011	0,0011	0,0026	0,0026	0,0024	0,0024	0,0023	0,0024
Brain	0,0003	0,0005	0,0005	0,0005	0,0011	0,0011	0,0011	0,0011	0,0010	0,0011
Breasts	0,0089	0,0113	0,0114	0,0113	0,0122	0,0126	0,0122	0,0125	0,0116	0,0119
Gallbladder Wall	0,0000	0,0000	0,0000	0,0000	0,0000	0,0000	0,0000	0,0000	0,0000	0,0000
Lower large intestine wall	0,0280	0,0375	0,0379	0,0369	0,0417	0,0404	0,0400	0,0408	0,0373	0,0399
Small Intestine	0,0007	0,0009	0,0009	0,0008	0,0019	0,0019	0,0018	0,0019	0,0017	0,0018
Stomach Wall	0,0739	0,0552	0,0404	0,0402	0,0447	0,0457	0,0417	0,0443	0,0400	0,0415
Upper large intestine wall	0,0008	0,0009	0,0009	0,0009	0,0020	0,0019	0,0018	0,0019	0,0017	0,0018
Heart Wall	0,0000	0,0000	0,0000	0,0000	0,0000	0,0000	0,0000	0,0000	0,0000	0,0000
Kidneys	0,0022	0,0018	0,0020	0,0020	0,0042	0,0044	0,0036	0,0043	0,0044	0,0043
Liver	0,0565	0,0538	0,0428	0,0442	0,0604	0,0608	0,0474	0,0483	0,0476	0,0472
Lungs	0,0520	0,0784	0,0748	0,0732	0,0638	0,0802	0,0814	0,0804	0,0777	0,0713
Muscle	0,0006	0,0007	0,0007	0,0007	0,0015	0,0015	0,0014	0,0014	0,0013	0,0014
Ovaries	0,0477	0,0648	0,0660	0,0644	0,0729	0,0707	0,0697	0,0712	0,0652	0,0695
Pancreas	0,0026	0,0017	0,0011	0,0011	0,0025	0,0025	0,0022	0,0024	0,0021	0,0022
Red Marrow	0,0704	0,0946	0,0702	0,0595	0,0746	0,0644	0,0692	0,0638	0,0576	0,0643
Osteogenic Cells	0,0044	0,0060	0,0052	0,0047	0,0056	0,0051	0,0053	0,0051	0,0047	0,0051
Skin	0,0014	0,0018	0,0018	0,0018	0,0020	0,0020	0,0019	0,0020	0,0019	0,0020
Spleen	0,3070	0,1340	0,0244	0,0224	0,0046	0,0055	0,0033	0,0046	0,0026	0,0027
Testes	0,0000	0,0000	0,0000	0,0000	0,0000	0,0000	0,0000	0,0000	0,0000	0,0000
Thymus	0,0005	0,0007	0,0007	0,0007	0,0015	0,0016	0,0016	0,0016	0,0015	0,0015
Thyroid	0,0079	0,0107	0,0115	0,0148	0,0136	0,0161	0,0128	0,0131	0,0150	0,0146
Urinary Bladder	0,0089	0,0126	0,0140	0,0140	0,0156	0,0154	0,0151	0,0156	0,0143	0,0152
Uterus	0,0006	0,0007	0,0008	0,0008	0,0018	0,0017	0,0017	0,0017	0,0016	0,0017
**Whole Body**	**0,6767**	**0,5700**	**0,4090**	**0,3960**	**0,4308**	**0,4381**	**0,4175**	**0,4205**	**0,3932**	**0,4032**
