# Supplementary material for: Tumour targeting and radiation dose of radioimmunotherapy with 90Y-rituximab in CD20+ B-cell lymphoma as predicted by 89Zr-rituximab immuno-PET: impact of preloading with unlabelled rituximab
Source: Eur J Nucl Med Mol Imaging. 2015 Mar 20;42(8):1304–14. doi: 10.1007/s00259-015-3025-6 (PMC4480335; doi:10.1007/s00259-015-3025-6)
Supplement: Supplementary file 4 — (PDF 51 kb) [file 259_2015_3025_MOESM4_ESM.pdf]

Supplementary Table 4 Absorbed Dose [mGy/MBq] 90Y-rituximab (organs)

	Without predosing					With predosing				
	1	2	3	4	5	1	2	3	4	5
Adrenals	0,167	0,254	0,309	0,315	0,346	0,345	0,336	0,355	0,323	0,340
Brain	0,167	0,254	0,309	0,315	0,346	0,345	0,336	0,355	0,323	0,340
Breasts	0,167	0,254	0,309	0,315	0,346	0,345	0,336	0,355	0,323	0,340
Gallbladder Wall	0,167	0,254	0,309	0,315	0,346	0,345	0,336	0,355	0,323	0,340
Lower large intestine wall	0,167	0,254	0,309	0,315	0,346	0,345	0,336	0,355	0,323	0,340
Small Intestine	0,167	0,254	0,309	0,315	0,346	0,345	0,336	0,355	0,323	0,340
Stomach Wall	0,167	0,254	0,309	0,315	0,346	0,345	0,336	0,355	0,323	0,340
Upper large intestine wall	0,167	0,254	0,309	0,315	0,346	0,345	0,336	0,355	0,323	0,340
Heart Wall	0,167	0,254	0,309	0,315	0,346	0,345	0,336	0,355	0,323	0,340
Kidneys	1,100	1,160	2,140	2,260	2,160	2,240	1,750	2,290	2,540	2,370
Liver	2,680	2,460	1,920	2,010	2,860	2,820	2,110	2,190	2,180	2,090
Lungs	0,868	2,110	2,110	2,050	1,460	2,160	2,350	2,240	2,230	1,920
Muscle	0,167	0,254	0,309	0,315	0,346	0,345	0,336	0,355	0,323	0,340
Ovaries	0,167	0,254	0,309	0,315	0,346	0,345	0,336	0,355	0,323	0,340
Pancreas	0,167	0,254	0,309	0,315	0,346	0,345	0,336	0,355	0,323	0,340
Red Marrow	1,890	2,730	1,790	1,390	1,880	1,450	1,730	1,450	1,280	1,540
Osteogenic Cells	1,380	2,000	1,470	1,220	1,570	1,290	1,460	1,300	1,160	1,340
Skin	0,167	0,254	0,309	0,315	0,346	0,345	0,336	0,355	0,323	0,340
Spleen	51,000	20,600	3,090	2,620	2,780	3,400	1,660	2,760	1,140	1,160
Testes	0,158	0,197	0,315	0,197	0,342	0,775	0,526	0,302	0,131	0,434
Thymus	0,167	0,254	0,309	0,315	0,346	0,345	0,336	0,355	0,323	0,340
Thyroid	0,172	0,172	0,196	0,564	0,368	0,589	0,245	0,245	0,564	0,466
Urinary Bladder	0,167	0,254	0,309	0,315	0,346	0,345	0,336	0,355	0,323	0,340
Uterus	0,167	0,254	0,309	0,315	0,346	0,345	0,336	0,355	0,323	0,340
