# Supplementary material for: Tumour targeting and radiation dose of radioimmunotherapy with 90Y-rituximab in CD20+ B-cell lymphoma as predicted by 89Zr-rituximab immuno-PET: impact of preloading with unlabelled rituximab
Source: Eur J Nucl Med Mol Imaging. 2015 Mar 20;42(8):1304–14. doi: 10.1007/s00259-015-3025-6 (PMC4480335; doi:10.1007/s00259-015-3025-6)
Supplement: Supplementary file 5 — (PDF 56 kb) [file 259_2015_3025_MOESM5_ESM.pdf]

	Supplementary Table 5 Effective Dose [mSv/MBq] 90Y-rituximab (whole body)									
	Without predosing					With predosing				
	1	2	3	4	5	1	2	3	4	5
Adrenals	0,0004	0,0006	0,0008	0,0008	0,0017	0,0009	0,0017	0,0009	0,0008	0,0009
Brain	0,0004	0,0006	0,0008	0,0008	0,0017	0,0009	0,0017	0,0009	0,0008	0,0009
Breasts	0,0084	0,0127	0,0155	0,0158	0,0173	0,0173	0,0168	0,0177	0,0161	0,0170
Gallbladder Wall	0,0000	0,0000	0,0000	0,0000	0,0000	0,0000	0,0000	0,0000	0,0000	0,0000
Lower large intestine wall	0,0201	0,0305	0,0371	0,0378	0,0415	0,0414	0,0403	0,0425	0,0388	0,0408
Small Intestine	0,0004	0,0006	0,0008	0,0008	0,0017	0,0009	0,0017	0,0009	0,0008	0,0009
Stomach Wall	0,0201	0,0305	0,0371	0,0378	0,0415	0,0414	0,0403	0,0425	0,0388	0,0408
Upper large intestine wall	0,0004	0,0006	0,0008	0,0008	0,0017	0,0009	0,0017	0,0009	0,0008	0,0009
Heart Wall	0,0000	0,0000	0,0000	0,0000	0,0000	0,0000	0,0000	0,0000	0,0000	0,0000
Kidneys	0,0028	0,0029	0,0054	0,0057	0,0108	0,0056	0,0088	0,0057	0,0636	0,0593
Liver	0,1340	0,1230	0,0962	0,1010	0,1430	0,1410	0,1050	0,1090	0,1090	0,1050
Lungs	0,1040	0,2530	0,2530	0,2460	0,1760	0,2600	0,2820	0,2680	0,2670	0,2300
Muscle	0,0004	0,0006	0,0008	0,0008	0,0017	0,0009	0,0017	0,0009	0,0008	0,0009
Ovaries	0,0335	0,0509	0,0000	0,0630	0,0691	0,0000	0,0000	0,0709	0,0646	0,0000
Pancreas	0,0004	0,0006	0,0008	0,0008	0,0017	0,0009	0,0017	0,0009	0,0008	0,0009
Red Marrow	0,2270	0,3270	0,2150	0,1660	0,2260	0,1740	0,2070	0,1740	0,1540	0,1850
Osteogenic Cells	0,0138	0,0200	0,0147	0,0122	0,0157	0,0129	0,0146	0,0130	0,0116	0,0134
Skin	0,0017	0,0025	0,0031	0,0032	0,0035	0,0035	0,0034	0,0036	0,0032	0,0034
Spleen	1,2700	0,5140	0,0772	0,0654	0,0139	0,0849	0,0083	0,0691	0,0029	0,0029
Testes	0,0000	0,0000	0,0631	0,0000	0,0000	0,1550	0,1050	0,0000	0,0000	0,0867
Thymus	0,0004	0,0006	0,0008	0,0008	0,0017	0,0009	0,0017	0,0009	0,0008	0,0009
Thyroid	0,0086	0,0086	0,0098	0,0282	0,0184	0,0294	0,0123	0,0123	0,0282	0,0233
Urinary Bladder	0,0084	0,0127	0,0155	0,0158	0,0173	0,0173	0,0168	0,0177	0,0161	0,0170
Uterus	0,0004	0,0006	0,0008	0,0008	0,0017	0,0009	0,0017	0,0009	0,0008	0,0009
**Whole Body**	**1,8556**	**1,3934**	**0,8489**	**0,8041**	**0,8078**	**0,9906**	**0,8740**	**0,8531**	**0,8203**	**0,8314**
